# Development of High Content Imaging Methods for Cell Death Detection in Human Pluripotent Stem Cell-Derived Cardiomyocytes

**DOI:** 10.1007/s12265-012-9396-1

**Published:** 2012-08-16

**Authors:** Maxime Mioulane, Gabor Foldes, Nadire N. Ali, Michael D. Schneider, Sian E. Harding

**Affiliations:** National Heart and Lung Institute, Imperial College, ICTEM, 4th Floor, Hammersmith Campus, Du Cane Rd, London, W12 0NN UK

**Keywords:** Stem cell, Methods, Cardiomyocytes apoptosis, Necrosis, Automated assay, High content microscopy

## Abstract

Human pluripotent stem cell-derived cardiomyocytes (hPSC-CM) are being investigated as a new source of cardiac cells for drug safety assessment. We developed a novel scalable high content microscopy-based method for the detection of cell death in hPSC-CM that can serve for future predictive in vitro cardio-toxicological screens. Using rat neonatal ventricular cardiomyocytes (RVNC) or hPSC-CM, assays for nuclear remodelling, mitochondrial status, apoptosis and necrosis were designed using a combination of fluorescent dyes and antibodies on an automated microscopy platform. This allowed the observation of a chelerythrine-induced concentration-dependent apoptosis to necrosis switch and time-dependent progression of early apoptotic cells towards a necrotic-like phenotype. Susceptibility of hPSC-CM to chelerythrine-stimulated apoptosis varied with time after differentiation, but at most time points, hPSC-CM were more resistant than RVNC. This simple and scalable humanized high-content assay generates accurate cardiotoxicity profiles that can serve as a base for further assessment of cardioprotective strategies and drug safety.

**Electronic supplementary material:**

The online version of this article (doi:10.1007/s12265-012-9396-1) contains supplementary material, which is available to authorized users.

## Introduction

Differentiation protocols for production of cardiomyocytes from human embryonic or induced pluripotent stem cells (hESC, hiPSC) are now robust, with staged application of growth factors in a monolayer system as an alternative to embryoid body methodology. It is possible to purchase these cardiomyocytes (hESC-CM, hiPSC-CM) from commercial sources, some with purities increased to more than 95 % by genetic selection. This has led to their increasing adoption by the pharmaceutical industry for drug discovery or toxicology testing with hopes that this humanised platform will improve predictive capabilities, since cardiotoxicity is one of the most common causes for attrition of both cardiac and non-cardiac drugs. There has also been uptake from academic researchers seeking an alternative to the standard rat/mouse primary adult or neonatal cells. A further advantage is the possibility to scale-up cell production and therefore increase assay throughput using platforms which can then be directly related or transferred to industry contexts. The plans in progress by both academic centres and industry worldwide to generate hiPSC lines from patients with known mutations are set to create an enormous resource for the researcher, which is likely to increase interest further. There have been striking successes in modelling the ion channel mutations in various clinical syndromes, with several long QT and other models described [13, 17, 19, 23]. The hiPSC-CM from these patients show not only the prolongation of action potential duration, observable despite the theoretical concern about mixed atrial/ventricular/nodal populations, but emergent characteristics such as afterdepolarisations and arrhythmias. Most recently, hiPSC-CM from patients with a troponin mutation were found to display βAR changes normally associated with acquired heart failure, in addition to sarcomeric changes, and to be rescued by beta-blockers or SERCA2a gene therapy [30].

For other processes including those involving complex signal transduction cascades, such as hypertrophy, proliferation and cell death, it has not been so clear whether the immature phenotype of pluripotent stem cell-derived cardiomyocytes will adequately model the terminally differentiated primary ventricular myocyte. Efforts are now underway to understand the characteristics of these changes in the pluripotent stem cell-derived cardiomyocytes and whether they utilise the authentic pathways observed in adult myocardium. We have previously shown that hypertrophic changes can be modelled in hESC-CM and are mediated by canonical kinase signalling cascades [5]. In this paper, we describe a similar strategy to study cell death in hESC-CM and hiPSC-CM (together termed hPSC-CM), with direct comparisons to rat neonatal ventricular cardiomyocyte (RNVC) preparation. Rodent neonatal cardiomyocyte preparations are presently the most widely used for investigation of longer-term cardiac signalling because their ability to survive in culture and to be manipulated genetically is favourable compared to the adult ventricular myocyte. However, as well as having the advantage of a human genotype, hPSC-CM remain stable in culture for considerably longer still (for more than a year) and are equal or superior in transfection efficiency to rodent neonatal cells. To take advantage of the scale-up possibilities, we have used a high content microscopy platform and optimised the assays to create a robust readout for early and late apoptosis and necrosis. This will provide a basis for studies of cardiotoxicity and protection, with a direct applicability in drug discovery and toxicology.

## Methods

### Cell Culture and Drug Treatment

Human ESC-CM were produced from the H7 hESC line (WiCell Inc). Cells were grown in the undifferentiated state on Matrigel (BD Biosciences) in mouse embryonic fibroblast conditioned medium, supplemented with 8 ng/ml of recombinant human FGF-2 (R&D Systems). Cardiac differentiation was performed using dense monolayers of hESC treated with 100 ng/ml Activin-A for 24 h and 10 ng/ml BMP-4 for 4 days in serum-free media, according to a previously described protocol [12]. Following induction with the growth factors, cultures were fed every other day with RPMI with 2 % B27. Spontaneously beating areas comprising sarcomeric myosin heavy chain- (MHC-) positive cells typically appeared after 15 days. Cardiomyocytes were isolated using trypsin for 3 to 5 min and collagenase IV (200 U/ml) for another 10 min. Clusters enriched in cardiomyocytes were triturated with a micro-pipette, and single cells were plated onto gelatinized 96 well plates. Human iPSC-CM were purchased from Cellular Dynamics International. These hiPSC-CM express mRFP and blasticidin resistance under the αMHC promoter and are purified before shipping (typically 95 % pure cardiomyocyte cultures). RNVC were isolated as described elsewhere [18]. Human ESC-CM, hiPSC-CM and RNVC were plated at 50,000 cells/cm^2^. Three days post-isolation or recovery, cells were treated with doxorubicin for 24 h or chelerythrine for 1.5 h in RPMI + B27 media.

### Live Staining and Immunochemistry

Live staining was performed in pure cardiomyocyte populations (i.e. RNVC and hiPSC-CM). Following drug treatment, cells were incubated with Vybrant® FAM caspases 3 and 7 (Invitrogen) for 1 h and stained with TMRM, ToPRO-3 and Hoechst 33342 (all Invitrogen) for 10 min. After washing with PBS and replacement with fresh media, stained cultures were placed in the Cellomics chamber at 37 °C/5 % CO_2_ and scanned. Alternatively, cardiomyocytes including hESC-CM were stained with BOBO-1 and Hoechst 33342 for 10 min prior to fixation with 4 % paraformaldehyde. Following permeabilization with 0.2 % Triton-X, cells were immunostained with polyclonal rabbit anti-caspase 3 (Abcam, 1:500) and monoclonal mouse anti-myosin heavy chain α/β (MHC α/β, clone 3–48, Abcam, 1:200) and anti-mouse Alexa 546 and anti-rabbit Alexa 647 (Invitrogen). Primary antibodies were detected with Alexa 546 and Alexa 647-conjugated secondary antibodies (1:400).

### Automated High Content Microscopy and Analysis

Images were acquired on ArrayScan™ VTi automated microscopy and image analysis system (Cellomics Inc., Pittsburgh, PA, USA) with ×10 objective suitable for filter sets. Cells were identified with Hoechst fluorescence that defined the nuclear area. Fluorescence intensities for the other channels were measured exclusively in the nucleus for Hoechst, ToPRO-3 and BOBO-1, in the perinuclear area for TMRM and MHC and in both cellular compartments for capsase-3 and caspase 3 and 7 substrate. Using modified SpotDetector Bioapplication, only high fluorescence intensities were detected and subsequent areas measured. In order to reduce false positive due to small but bright autofluorescent particles or to background noise, we used the *spotdetection* tool that recognizes contiguous pixels with high intensity, and we discarded spots with smaller size. Cardiomyocytes were identified using anti-MHC antibody. The fluorescence intensity threshold discriminating cardiomyocytes and non-cardiomyocytes was set manually in each experiment. In mixed cultures of hESC-CM, data were collected only in MHC positive cells. For caspases, a significant basal level in healthy cells complicated the analysis, and the method of thresholding is described further in the “Results” section. For other markers, discrimination between live and dead cells was not based on a predetermined fluorescence intensity because slight variation in quality of the staining or in culture conditions (cell density) makes the use of a fixed threshold inappropriate across experiments. Instead, we assumed that the rate of cell death in control conditions resulting from normal cell turnover is reasonably consistent. Supported by thorough image observations and data from others [11, 26], we made the assumption that 5 % of control cells were either dead or in the process of dying. To set the threshold above zero also gave the possibility for protective effects to be detected under control conditions. Nuclear shape (using numeric descriptors of shape complexity ObjectShapeP2A) is an index based on the ratio of the length and the width. Healthy cells are typically circular or slightly elongated with a small nuclear shape index whereas dying cells that undergo nuclear fragmentation may not only be bigger (high Hoechst area) but may exhibit altered nuclear shape. For TMRM, active extrusion of the dye occurs in healthy cells, and these living TMRM negative cells confound with mitochondrial-compromised cells [9]. We made the assumption that increase in the TMRM negative population in treated cells was exclusively due to increase in mitochondrial dysfunction and not to increased extrusion of the fluorescent dye. For (1) a given individual parameter, (2) different composite categories (e.g. late apoptosis) or (3) total cell death the results are expressed as an index, calculated as: (% positive − threshold)/(100 % − threshold).

### Statistics

Results are expressed as mean ± SEM. Paired or unpaired *t* tests or one-way ANOVA were used as appropriate. Differences at the level of *P* < 0.05 were considered statistically significant.

## Results

Early events in the apoptotic pathway include loss of mitochondrial membrane potential and activation of caspases, followed by changes in cell shape with membrane blebbing and detachment, then later events such as nuclear remodelling and final loss of sarcolemmal integrity [29]. We will describe the markers used for these events and their optimisation for the Cellomics Arrayscan™ high content screening platform. Examples from neonatal and hESC or hiPSC-derived cardiomyocytes are given for the optimisation steps, and a direct comparison of sensitivity of the different cell types to cell stressor chelerythrine is made under the chosen conditions.

### Mitochondrial Membrane Potential

**Fig. 1:**
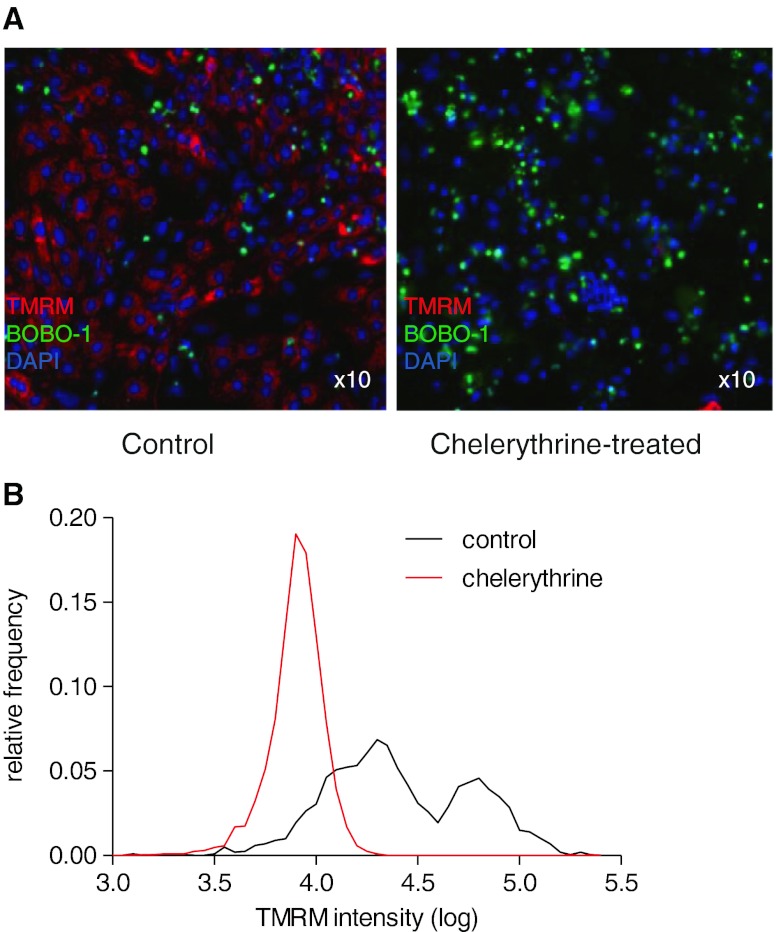
Mitochondrial membrane potential collapse during chelerythrine-induced cell death in rat neonatal ventricular myocytes. Rat neonatal ventricular myocytes were exposed to chelerythrine (100 μM) in order to induce maximal cell damage and complete loss of the mitochondrial membrane potential. **a** In control cultures (*left panel*), most cells were positive for TMRM (*red*); nuclei were identified by DAPI (*blue*). In chelerythrine-treated myocytes (*right panel*), the TMRM signal disappeared and the proportion of permeable cells (BOBO-1, *green*) was increased. **b** An example experiment (2,000 cells) showing the distribution of fluorescence intensity of TMRM in cells in the presence and without chelerythrine. In chelerythrine-treated cells, where mitochondrial membrane potential is lost, the intensity graph does not overlap with low TMRM peak in control

Dissipation of the mitochondrial membrane potential (*m*∆ψ) initiates cell death in cardiomyocytes [16]. During apoptosis, mitochondrial outer membrane permeabilisation (MOMP) is necessarily for apoptosome formation and thus precedes effector caspase activation (although MOMP-independent caspase 3 activation has also been described [7]). We used the lipophilic cationic potentiometric dye tetramethylrhodamine, methyl ester (TMRM) as a sensor of *m*∆ψ for live cells. Neonatal RVNC stained with TMRM exhibit bi-modal fluorescence intensity distribution with a large population of low TMRM staining in control (Fig. 1). In addition to the small subset of apoptotic and necrotic cells that are TMRM negative, active extrusion of the dye through drug-resistance pump can explain the large number of cells with low TMRM [9]. Treatment with the apoptosis/necrosis inducer chelerythrine (100 μM) induced complete loss of TMRM signal in both low and high intensity cells (Fig. 1), and this was complete after 160-min exposure.

Live cell dyes such as TMRM work well for pure populations such as RNVC or genetically purified stem cell-derived cardiomyocytes (such as the CDI hiPSC-CM) but are leached during fixation. For the mixed populations obtained from most differentiation procedures, the use of reporters such as Mitotracker (which is retained during processing) and co-staining with a cardiac marker such as troponin I (TnI) or αMHC is required to localise signal to cardiomyocytes. Supplementary figure 1 shows the algorithm for cardiomyocyte detection on the Cellomics Arrayscan platform. This identifies cells from nuclear staining and quantifies MHC staining intensity in a perinuclear ring. Most nuclei are discreet, but there are small numbers where more than one nucleus appears to be captured in a circle. These can be fragments, as discussed in the cell death section. They can also be dividing cells or true binucleated cardiomyocytes (which is the normal adult form). These are counted as single cells, and we estimate an event rate of <20 % [5].

### Activation of Effector Caspases

**Fig. 2:**
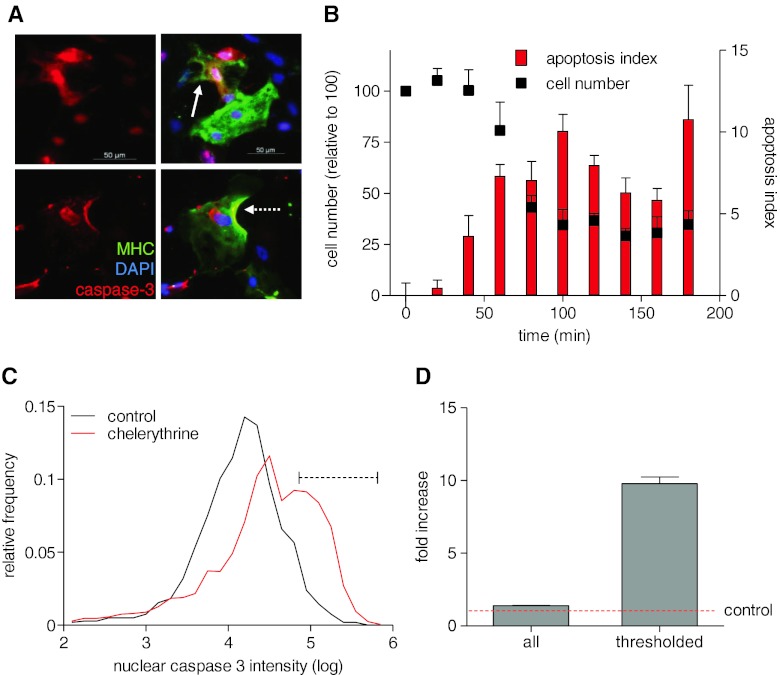
Activation of caspase 3 in cardiomyocytes. **a** Human ESC-CM are identified by immunostaining in fixed cultures, using anti-MHC antibody (*green*) as a cardiac-specific marker. Active caspase 3 (*red*) is most visible in the nuclear and perinuclear regions of the apoptotic cells. Activation of caspases is typically accompanied by cell shrinkage and detachment (*solid* and *dotted white arrows*, respectively). **b** Activation of caspase 3 and change in cell number in hESC-CM treated with chelerythrine. Human ESC-CM were exposed to 10 μM chelerythrine, and apoptosis index was measured at different time points. Increase in apoptosis is accompanied with a dramatic reduction in cell number due to cell detachment (*n* = 6 from one experiment, 2,000 cells per well). Apoptosis index was calculated as: (% positive − threshold)/(100 % − threshold). **c** hESC-CM double-stained for caspase 3 and MHC; graph shows data from 5,270 control and 1,110 chelerythrine-treated cells. **d** Fold changes between control and chelerythrine-treated values using either unthresholded results (all) or the values remaining after removing those below the threshold (thresholded)

Activation of effector caspases (caspases 3, 6 and 7) is key in the dismantling of the cell structure and machinery during apoptosis [32]. Mitochondrial or receptor-mediated apoptosis both induce a cascade of caspase activation that converges ultimately to the conversion of the pro-caspase 3 into proteolytically active caspase 3. Assessment of caspase 3 activity can be performed by measurement of caspase 3 (and caspase 7)-mediated degradation of specific fluorescent substrate. Alternatively, anti-active caspase 3 antibody conjugated with a secondary antibody gives a measure of active caspase 3 content in the cell at the time of fixation. Stable control hESC-CM stained with anti-caspase 3 antibody show significant fluorescence, mostly localized in the nucleus, seen as sub-lethal levels of active caspase 3. Higher levels of anti-caspase 3, localised in the nuclear and peri-nuclear areas, were found in cells that underwent morphological changes typical of apoptosis such as cell shrinking and the start of detachment (Fig. 2a). In a time course experiment using chelerythrine, active caspase 3 increased rapidly with concomitant decrease in hESC-CM number in the culture (Fig. 2b). Active caspase 3 is present in hESC-CM at a significant level (Fig. 2c), but chelerythrine treatment produces a bimodal population intensity value distribution (note the logarithmic scale). Using a threshold value derived from normal populations increased the discrimination between conditions (Fig. 2c, d). Comparison of absolute values from the whole population gives a modest fold-induction (×1.4), but thresholding at mean ± 1.5× SD of control gives better discrimination, with a 9.8-fold difference from control. Calculating the *Z*′ of this assay with this thresholding gives an acceptable value of 0.81. Simulations of other threshold values showed that a cutoff point excluding all control cells gives a maximum *Z*′ of 0.85, which is similarly excellent but would exclude many caspase-high cells in the second population and potentially limit detection when apoptotic changes were small.

### Relation Between Mitochondrial Membrane Potential and Caspase Activation

Co-staining of hiPSC-CM with TMRM and caspase 3 and 7 substrate gave a different cell death profile after exposure to 10 μM chelerythrine or 10 μM doxorubicin. Chelerythrine induced strong mitochondrial dysfunction whereas doxorubicin did not. A high proportion (66 ± 7.5 %) of apoptotic cells (caspase 3 and 7 positive cells) underwent loss in TMRM signal in chelerythrine treated cells. On the other hand, only 4 ± 0.8 % of hiPSC-CM were both caspase positive and TMRM negative when treated with doxorubicin suggesting mitochondrial-independent activation of caspases 3 and 7 (doxorubicin vs. chelerythrine *p* < 0.001, *n* = 22 and *n* = 12, respectively). Therefore, apoptotic cells were characterized according to their mitochondrial status: apoptosis with MOMP and apoptosis without MOMP.

### Cell Membrane Permeability

**Fig. 3:**
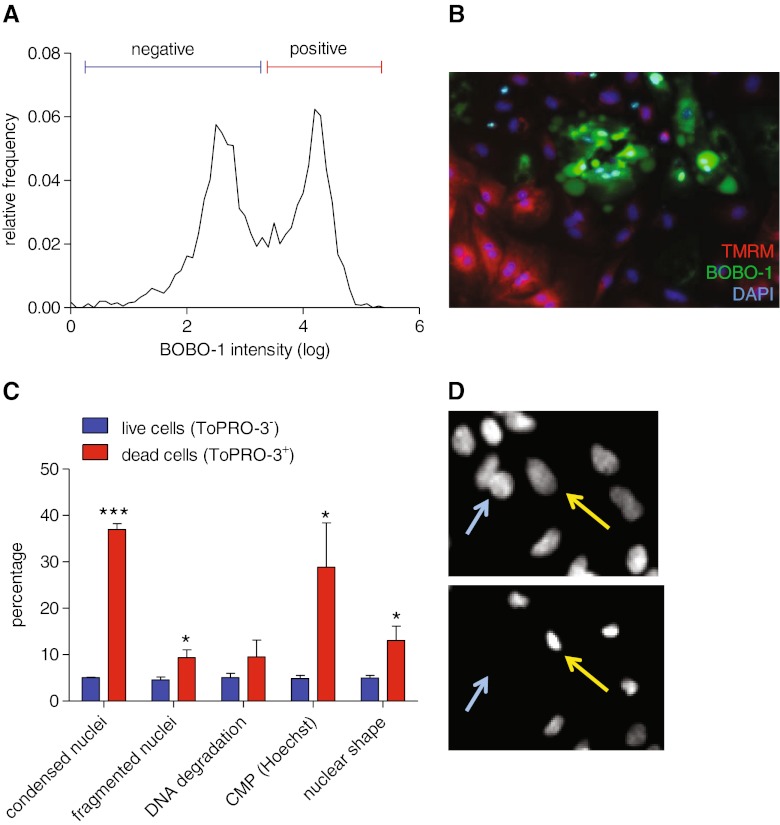
Nuclear events during cell death in human induced pluripotent stem cell-derived cardiomyocytes**. a** Bimodal distribution of nuclear BOBO-1 fluorescence intensity. **b** Healthy cells show intact mitochondria with TMRM positivity whereas dead cells are exclusively BOBO-1-positive and TMRM-negative. **c** Nuclear remodelling in ToPRO-3-negative and -positive hiPSC-CM under control conditions. The subset of dead cardiomyocytes in control were assessed for their nuclear size, DNA content, membrane permeabilisation (CMP, high Hoechst intensity) and nuclear fragmentation (*n* = 5 preparations). **d** Rat neonatal ventricular myocytes exposed to chelerythrine at t0 and t180 min (repeated images from the same field). Nuclei are large and round shaped before the drug induces toxic effects, whereas nuclei are rather small, with condensed chromatin after 3 h of exposure (*yellow arrows*). Alternatively, cells can detach from the substrate, resulting in reduction in cell number (*blue arrow*). Note that pyknotic nuclei are usually brighter as a consequence of concomitant cell membrane permeabilisation (CMP/Hoechst) or extreme condensation of the chromatin

At the later stage of apoptosis or following traumatic cell damage leading to necrosis, cells are no longer able to maintain the integrity of their membrane. This results in solute equilibrium between the intracellular and extracellular milieu, arrest in metabolic activity and thus characterizes the final stage in the cell death process [33]. Impermeant dyes such as commercialized cyanin dimer or propidium iodine are able to enter dead cells and exhibit strong fluorescence when bound to nucleic acids. Here, because of fluorescence channel availability or requirement for further fixation steps, we used different cyanine dimer nucleic acid dyes such as BOBO-1 and ToPRO-3. These dyes differ in their light emission wavelength but have similar function and so were used interchangeably. Cells treated with doxorubicin for 24 h and stained with BOBO-1 showed, as expected, a bimodal distribution in nuclear average intensity (Fig. 3a). Note also the clear separation between BOBO-1 positive cells and those with high TMRM signal (Fig. 3b).

### Nuclear Events

Change in nuclear morphology is a hallmark of the final stage in cell death. In apoptosis, these include chromatin condensation, nuclear fragmentation and DNA degradation. In our assay, we recorded nuclear events as additional toxicity readouts regardless of the cell death pathway involved. Extreme nuclear sizes discriminated chromatin condensation (pyknosis) and nuclear fragmentation (karyorrhexis), although the potential for a fragmented nucleus to be read by the high content screening as an enlarged one required care in the analysis. Terminally apoptotic cells, which are expected to undergo nuclear remodelling due to the action of nuclear proteases, also allow the entry of the impermeant dye ToPRO-3 due to increase membrane permeability. Nuclear characteristics of ToPRO-3 positive cells differ clearly from ToPRO-3 negative cells (Fig. 3c). Dead cells have either smaller or bigger nucleus size than control because of condensed or fragmented nuclei respectively, or display the other changes shown in Fig. 3c. Hoechst intensity distribution is commonly used in cytometry and high content assays for cell cycle analysis. In cytometry studies, there may be a population of cells below the G1/0 or 2 N peak with lower Hoechst intensity seen as an apoptotic population with degraded DNA [1]. Conversely, facilitated penetration of Hoechst in terminal cells results in increased Hoechst nuclear intensity (cell membrane permeability (CMP)/Hoechst readout). Here, dead cells in control conditions exhibited nuclei with higher Hoechst intensity but not with lower intensity (Fig. 3c, d). This suggests that apoptosis-related DNA degradation does not naturally occur during normal cell turnover in culture. In contrast, DNA degradation is the most important nuclear event in doxorubicin-induced cell apoptosis (see below).

### Late Apoptosis Versus Necrosis

**Fig. 4:**
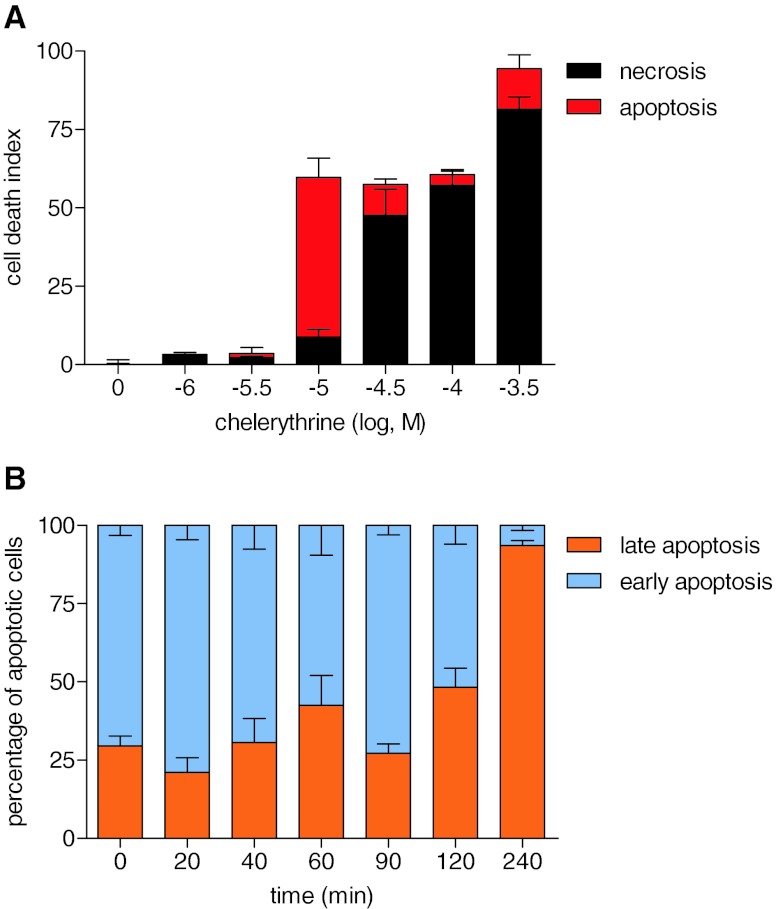
Concentration and time-dependent effects of chelerythrine in rat neonatal ventricular myocytes. **a** Chelerythrine dose-dependent induction of apoptosis and necrosis in RNVC. *Stacked bar graph* shows that apoptosis peaked at a concentration of 10 μM (90 min) whereas necrosis predominated at higher chelerythrine concentrations. *n* = 6 from 1 experiment, 2,000 cells analysed per well. **b** Caspase-3 activation is followed by secondary necrosis in RNVC. Cells were exposed to chelerythrine (10 μM) at different times and stained for CMP marker BOBO-1 and apoptotic marker anti-caspase 3. *Stacked bar graph* shows early apoptosis (caspase 3-positive/BOBO-1-negative cells) predominates during the first minutes of exposure with the drug whereas late apoptosis (caspase 3-positive/BOBO-1-positive cells) characterized the apoptotic population at a later time point (240 min). Cell death index was calculated as: (% positive − threshold)/(100 % − threshold)

**Fig. 5:**
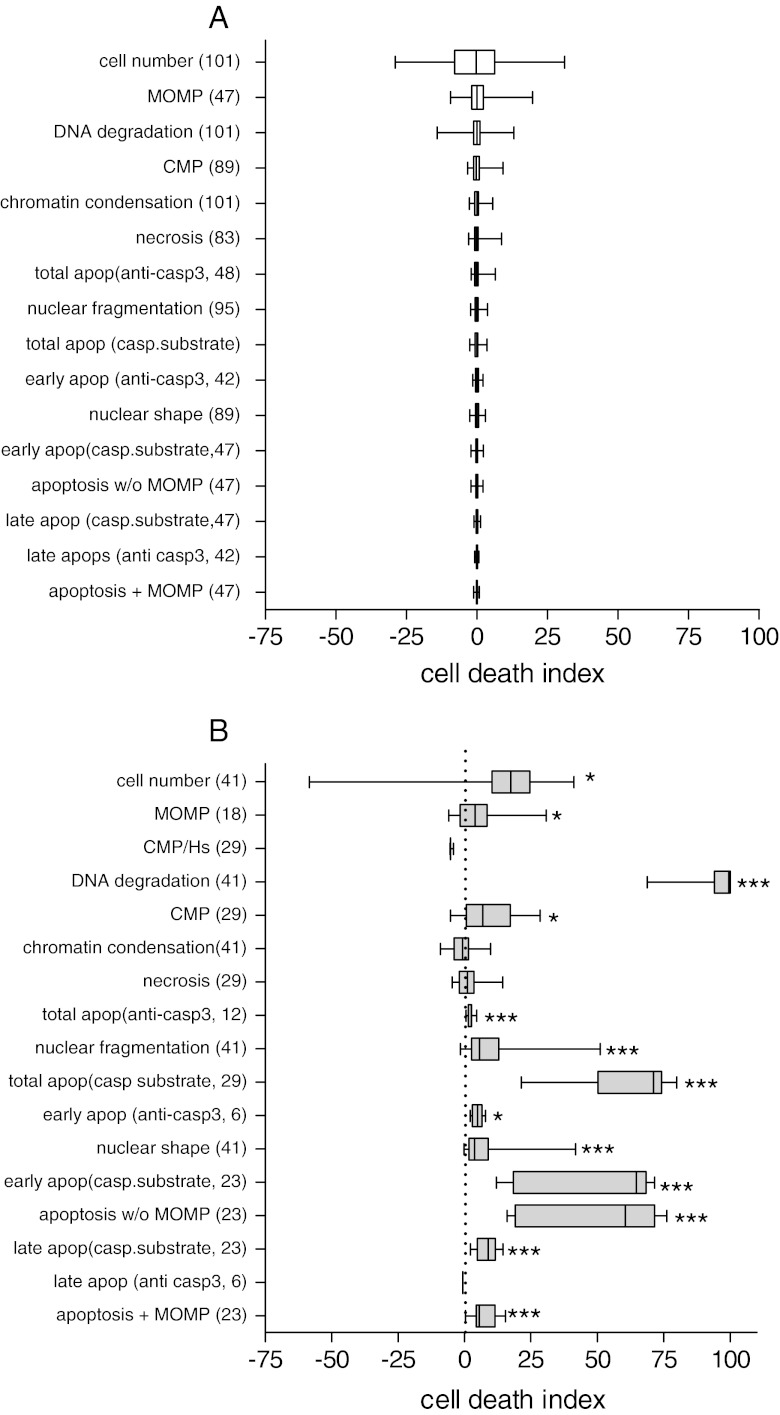
Individual and composite readouts to characterize cardiotoxicity profiles. Median, upper and lower quartiles and values are shown in **a** control and **b** doxorubicin-treated hiPSC-CM, with *n* = wells in *brackets*. Parameters were ranked by interquartile range of control populations. *CMP* cell membrane permeability, *Hs* Hoechst, *MOMP* mitochondrial outer membrane permeabilisation, *apop* apoptosis, *casp* caspase. *P < 0.05;**P < 0.01; ***P < 0.001 versus respective controls

Whether a cell undergoes apoptosis with activation of caspases or undergoes a less regulated death like necrosis depends largely on the drug action and the drug concentration. Chelerythrine is known to induce apoptosis at 10 μM and necrosis at higher concentration in RNVC [34]. We confirmed this concentration effect in RNVC using apoptosis as defined before and necrosis as CMP positive cells within the caspase 3 negative population (Fig. 4a). Finally, we confirmed the progression of apoptotic cells towards a necrotic-like phenotype in RNVC exposed to prolonged treatment with 10 μM chelerythrine. A majority of apoptotic cells were caspase 3 positive cells without CMP during the 90 first minutes whereas caspase 3^+^/CMP^+^ cells represented 50 and 95 % of the apoptotic population at 120 and 240 min of chelerythrine exposure, respectively (Fig. 4b). Therefore, we created early and late stage apoptosis categories to characterise the progression of cardiomyocytes towards the apoptotic process based on their CMP and caspase 3 status also for doxorubicin, please see Fig. 5.

### Overall Assessment of Death Profiles

**Fig. 6:**
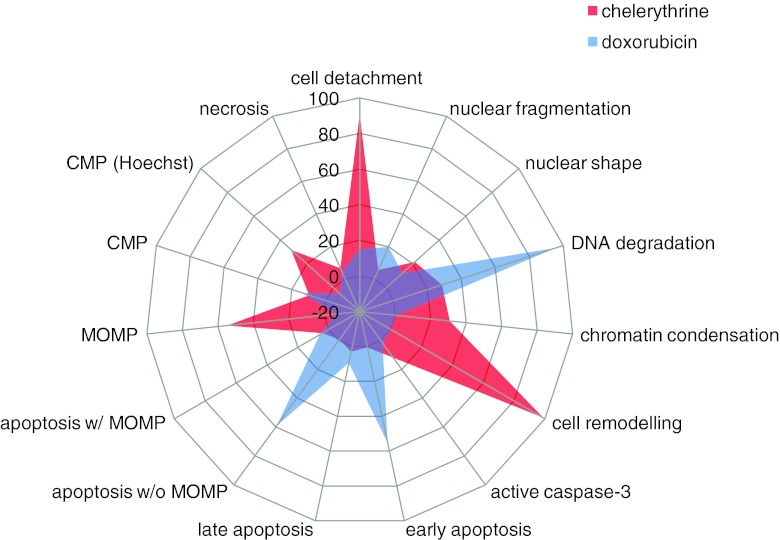
Radar chart showing multivariate chelerythrine and doxorubicin-induced cell death profiles in hiPSC-CM. Both drugs induced cardiomyocyte death but with clearly different toxicity signatures. Chelerythrine induced mitochondria dysfunction (*MOMP*), cell shape remodelling, chromatin condensation and moderate apoptosis and necrosis. Doxorubicin was a strong inducer of DNA degradation, early apoptosis (caspase 3 and 7 substrate without cell membrane permeabilization) with intact mitochondrial membrane potential. Axis shows cell death index, with zero representing the control. Doxorubicin: *n* = 22, four experiments; chelerythrine *n* = 12, two experiments

Individual (e.g. MOMP) and composite (e.g. late apoptosis) measures were ranked by variability both under control conditions and after challenge with 10 μM doxorubicin. Cell number was clearly variable simply because of differences in plating, but other parameters are expressed as a proportion of cells remaining. Individual parameters are more variable than composite and, in general, show less significant changes on doxorubicin treatment: This supports the use of composite measures. Further, a “fingerprint” representation of the cell death profile can be done for different stimuli, cell types and conditions. In Fig. 6, we show an example radar graph for doxorubicin and chelerythrine in which the different aspects of apoptosis and necrosis are represented.

### Comparison of Cell Death in RNVC and hESC-CM

**Fig. 7:**
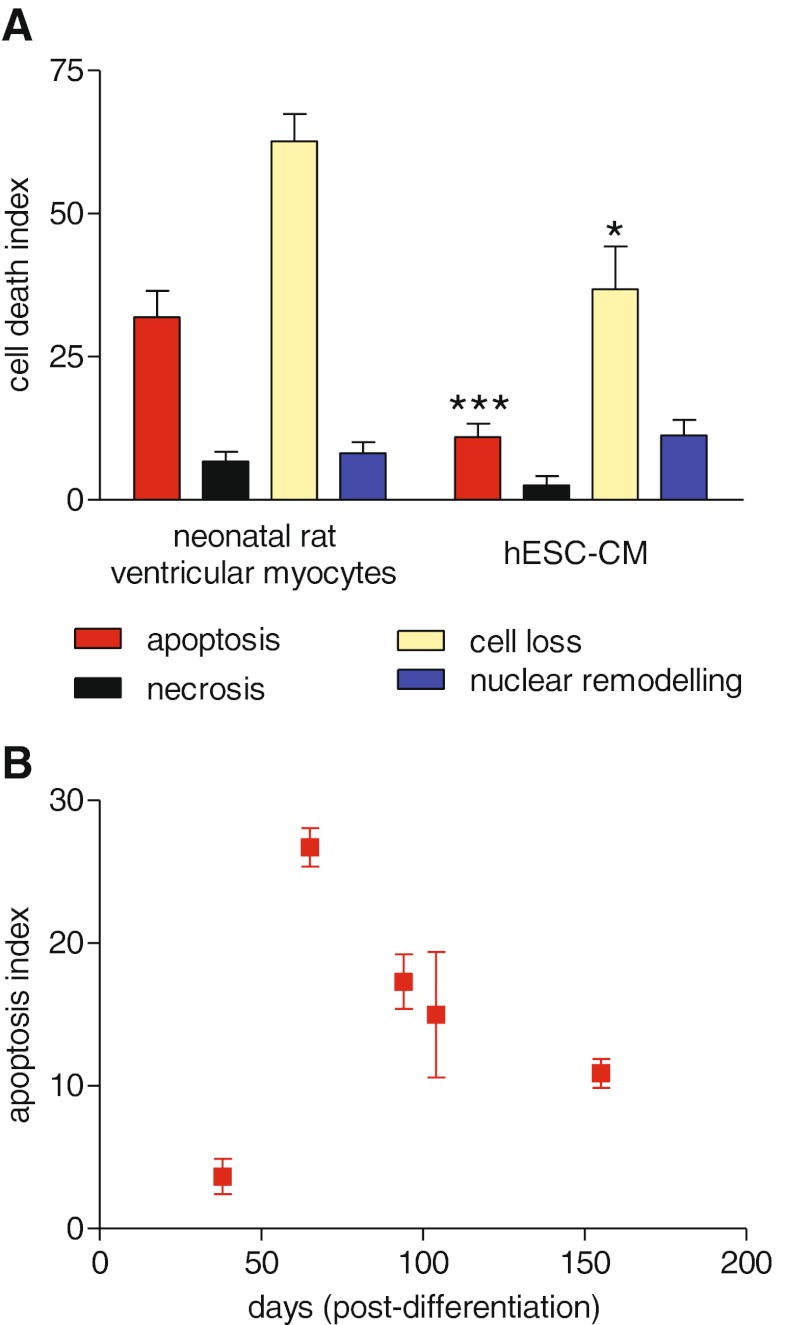
Chelerythrine induced a different cell death signature in hESC-CM and in rat neonatal ventricular myocytes. **a** Cardiomyocytes were exposed to 10 μM of chelerythrine for 90 min. Cell death index was calculated as: (% positive − threshold)/(100 % − threshold). RNVC (*left bars*) *n* = 11 from three experiments; hESC-CM (*right bars*) *n* = 21 from five experiments. Student’s *t* test, *p < 0.05, ***p < 0.001 compared to corresponding bar in the neonatal myocyte group. **b** Sensitivity to chelerythrine-induced apoptosis in hESC-CM varies according to the stage of differentiation. Apoptosis index is shown as mean ± SEM in hESC-CM. *n* = 3 for days 38 and 65; *n* = 6 for days 94, 104 and 155

Chelerythrine induced caspase 3 activation, nuclear remodelling, cell loss and moderate necrosis. Together this confirms a pro-apoptotic effect of chelerythrine in both cardiomyocyte models. However, comparison of the cell death indices revealed a significant quantitative difference between hESC-CM and RNVC in term of caspase 3 activation (apoptosis index = 11 ± 2.4 and 31.9 ± 4.6, respectively, *p* = 0.0001), a difference in cell loss (36.8 ± 7.5 and 62.6 ± 4.7, respectively *p* = 0.028) but no difference in necrosis and nuclear remodelling (*p* = 0.08 and *p* = 0.39, respectively) (Fig. 7). These data suggest that hESC-CM are more resistant than RNVC following chelerythrine treatment. Importantly, there was no switch from apoptosis to necrosis in hESC-CM as shown by the low and similar necrotic index in both species.

### Cell Death in hESC-CM with Time in Culture

We further investigated the effect of chelerythrine in hESC-CM at different times after differentiation to cardiomyocytes from 38 days, which represents an early stage hESC-CM in maturation, to 155 days, considered as a late stage [28]. Our data suggest that the sensitivity of hESC-CM to chelerythrine differs strongly according to the stage of differentiation (Fig. 5). Early hESC-CM were resistant to apoptosis but a rapid change occurred during the intermediate stage (day 65) where hESC-CM seemed almost as sensitive to chelerythrine as the RNVC. From the intermediate stage to the late stage of differentiation, hESC-CM developed progressive resistance to chelerythrine but were still more sensitive than the early stage.

## Discussion

High content screening (HCS) is now a central paradigm for drug discovery, including the investigation of off-targets effects such as cell toxicity. In addition to its promising use for industry, automated microscopy in micro-plate format has several advantages over other methods to interrogate hPSC-CM cell death. Firstly, the generation of hPSC-CM remains relatively expensive in comparison with immortalized cell lines so that the use of smaller cell numbers is an advantage. Secondly, although differentiation of hPSC can be directed into cardiovascular lineages, a proportion of the differentiated derivatives will not be of the cardiomyocyte phenotype, which excludes any method based on cell lysates. Even if mechanical and genetic purification methods are available and have been shown to be successful [2, 9], they are still technically challenging. There is also the theoretical concern that selection methods, such as strong antibiotic treatment (for cells with antibiotic resistance genes) or FACS sorting, have the potential to favour a subpopulation of hardy cells over those which are more fragile. This could bias the sensitivity of cell death responses in general. When mixed cultures must be used, immunochemical methods to identify hPSC-CM by cardiac-specific antibodies can be combined with other markers of the cell death process. Here we have described both live assays in pure cultures of cardiomyocytes and assays with fixed cells in mixed cultures.

The onset of the cell death program ranges from few seconds to several hours [24], whether the stimulus acts through the activation of pro-apoptotic genes or induces direct damage to organelles. Apart from the typical morphological changes specific to the different forms of cell death, there is no universal biochemical marker that identifies clearly an apoptotic or a necrotic cell. With a range of markers for mitochondrial membrane potential, caspase activation, cell loss, nuclear remodelling and cell membrane permeabilisation, we have defined the path through early and late apoptosis to necrosis.

Early events in the apoptosis program involve the mitochondrial translocation of pro-death molecules into the cytosol and the nucleus, leading to effector caspase activation and DNA damage, respectively [27]. The dissipation of the mitochondrial membrane potential is seen as central in the release of pro-death factors although release of cytochrome-c has been observed in the absence of mitochondrial membrane potential collapse [15]. The participation of mitochondrial dysfunction in cardiomyocyte apoptosis may have critical implications such as the depletion of the intracellular ATP stores, which orchestrates the apoptosis to necrosis switch. Here we show that hiPSC-CM exposed to doxorubicin can undergo effector caspase activation in the absence of mitochondrial membrane potential dissipation, which may have important implications for future cardioprotective strategies in cancer chemotherapies. However, our system does not allow investigation of other aspects of mitochondrial dysfunction, such as mitochondrial fragmentation, that may also occur during doxorubicin treatment [22].

The activation of caspase 3 is considered as an intermediate event in the cell death process as well as the onset of the irreversible dismantling of the cell machinery [4]. Importantly, necrotic cell death does not involve caspase proteolysis, and thus, the activation of caspases is a good candidate for the discrimination between the two forms of cell death [27]. However, active caspases, in particular caspase 3, can be involved in other processes than apoptosis, including cell division [8] and differentiation of tumour cells and embryonic stem cells [6, 14, 20, 31]. Furthermore, crosstalk between differentiation and death pathways was investigated [3], and caspase 3 has been demonstrated indispensable for normal cardiovascular formation in mouse [21]. This last point is particularly important as hESC-CM are immature cardiomyocytes freshly derived from hESC and may be in the process of phenotypic maturation. It is possible that the significant basal level of caspase 3 in hESC-CM that we observed is a characteristic of incompletely matured hESC-CM. However, the activation of caspase 3 is considered, to date, as the most reliable marker to detect apoptosis [4, 27]. Here we showed that active caspase 3-associated fluorescence intensity increased after exposure to 10 μM chelerythrine in hESC-CM and RNVC and that this was sufficiently differentiated from basal levels to give an assay with an acceptable *Z*′ score. Also, the observation of cells by fluorescent microscopy revealed that high level of caspase 3 fluorescence was associated with morphological changes typical of apoptosis (membrane blebbing, lifting and shrinkage). The activation of caspase 3 in either RNVC or hESC-CM was followed by a dramatic loss in cell number, as a result of cell detachment, and was accompanied by nuclear remodelling, mainly seen as chromatin condensation. Together, our data confirm that activation of caspase 3 as assessed by immunocytochemical methods is a good marker to distinguish apoptosis and necrosis in both primary and pluripotent stem cell-derived cardiomyocytes.

Cell detachment from the substrate, observed as cell loss, was similarly an intermediate event. Interestingly, it was neither correlated to the concentration of chelerythrine nor to the necrotic index but was strongly correlated to the apoptotic index. We showed that there was no overestimation of the cell number resulting from possible nuclear fragmentation but that apoptotic cells were more prone to detachment than the necrotic cells. Two hundred and eighty targets of caspase 3 have been identified so far, including structural proteins that maintain cell morphology and cell adhesion [4]. Proteolysis of cadherin and integrins by activated caspases can facilitate membrane detachment and subsequent cell loss [4]. In both apoptosis and necrosis, elevated intracellular calcium activates proteases such as calpain and lysosomal cathepsins [25], but the extent of the cytoskeleton and adhesion protein remodelling is likely to be more severe in apoptosis due to the additional proteolytic action of the caspase 3. However, while biologically consistent, cell loss can be a limiting factor for automated assays because of the inability to characterise the lost cardiomyocytes and the reduced power of the assay as cell numbers dwindle.

Cell permeabilisation was a late event in the cell death process. Depending on the secondary antibody fluorescence wavelength for MHC and caspase 3 detection, different impermeant dyes can be used to assess sarcolemmal integrity, such as BOBO-1 (green pseudocolour), propidium iodide (red) and ToPRO-1 (far red). BOBO-1, for example, discriminates clearly live and dead cells, giving a bi-modal distribution of intensity in non-fixed culture. We found that the different plasma membrane impermeant dyes correlate very well between each other (not shown) and thus can be alternatively used according to the experimental design. BOBO-1 and caspase 3 function as a couple to discriminate apoptosis from necrosis and to determine the stage in the progression of apoptosis. We created categories such as early apoptosis (high caspase/low BOBO-1), late apoptosis (high caspase/high BOBO-1) and necrosis (low caspase 3/high BOBO-1) and showed that these categories are relevant to the time scale of apoptosis and to the dose-dependent switch between programmed cell death and traumatic cell death using chelerythrine. Changes in nuclear morphology provided added information about the late stage of cell death, with either fragmentation or condensation of nuclei as well as alterations in Hoechst staining intensity being associated with cell death as determined by permeabilisation.

After using RNVC, hESC-CM or iPSC-CM to optimise the assay conditions and define the progression of markers, we performed a more explicit comparison between the primary and stem cell-derived cardiomyocytes. We used the concentration and the exposure time of chelerythrine that induced the maximal apoptotic effect in RNVC. Human ESC-CM showed a similar profile, with the peak of caspase 3 activation being at 10 μM and apoptosis switching to necrosis at greater doses. In both cell types, the effect started at 40 min and peaked at 90 min. However, at this maximal apoptotic concentration, early hESC-CM were more resistant than rat RNVC in terms of the apoptosis and cell loss. Importantly, we showed that this resistance to apoptosis was not offset by an increase necrosis in hESC-CM. Looking further, we saw that the sensitivity of chelerythrine-induced apoptosis changed with the stage of differentiation of hESC-CM. Early hESC-CM (day 38) show very little sensitivity to chelerythrine, but a rapid change occurred at the intermediate stage (day 65) where the sensitivity was maximal (26.7 ± 1.3) and similar to that of RNVC. Further hESC-CM aging/maturation brought a decreased sensitivity to chelerythrine as seen by the lower apoptosis index during the late stage (days 94, 105 and 155). Chelerythrine is a benzophenanthridine alkaloid commonly used in research as a potent selective protein kinase C (PKC) inhibitor [10], but in some studies, chelerythrine-induced apoptosis in rat neonatal cardiomyocytes was found to be mediated through the generation of intracellular ROS rather than PKC inhibition [34]. Immature cardiomyocytes are typically resistant to oxidative stress, which would account for the initial insensitivity of the hESC-CM to chelerythrine but makes the later re-development of resistance more puzzling. It would be interesting to investigate whether the peak of sensitivity to apoptosis in hESC-CM at the intermediate stage of differentiation mimics the developmental remodelling during morphogenesis of the embryonic heart, which is largely dependent upon caspase 3 activity, at least in animals [35]. Furthermore, this would be consistent with the increased resistance of late hESC-CM because the myocytes predisposed to undergo developmental-dependent apoptosis during the intermediate stage would die, resulting in a positive selection of resistant cardiomyocytes. Despite their convenience in the HCS system, this resistant phenotype would suggest that caution is needed when incorporating hPSC-CM into assays to predict toxicity in the adult human heart.

In summary, a portfolio of markers defining the time course and process of cell death can be used with either live or fixed pluripotent stem cell-derived cardiomyocytes. The statistical power of high content imaging is able to distinguish subtle differences in mechanism between toxic agents and to produce a “fingerprint” that can be either cell type or stimulus-specific. Composite end points such as late apoptosis or total cell death showed reduced variability compared to individual markers. Pluripotent stem cell-derived cardiomyocytes are well suited to this system because of their robust cardiac phenotype and stability in prolonged culture. For pharmaceutical companies, they could replace the CHO and HEK cells currently used for toxicity profiling. Although not replacing animals directly, their cardiomyocyte phenotype would allow a more sophisticated assessment of cardiotoxicity, as opposed to general cell toxicity, and therefore potentially remove a further tranche of unsuitable compounds before in vivo studies. For the researcher, the preparations most likely to be replaced are the adult or neonatal rodent cardiomyocytes, presently a mainstay for in vitro studies. The enthusiasm of the field for exploration of the potential of iPSC-derived cardiomyocytes is therefore justified, although the challenge of their immature characteristics persists, and their predictive power remains to be established. Their human origin will be an overriding advantage, especially with the advent of an era where a large number of genotype-specific iPSC lines will soon become available to the cardiac researcher.

## Electronic supplementary material

Below is the link to the electronic supplementary material.

Supplementary Figure 1 Cellomics algorithm for the detection of MHC-positive hESC-CM. **A** DAPI (*blue*) identifies cell nuclei and MHC (*green*) defines cardiomyocytes in differentiated hESC cultures. **B** The bioassay algorithm detects each nucleus (*blue inner outline* for accepted nucleus) and thus delineates an inner region corresponding to the nucleus and an outer region called ring that applies from the peri-nuclear area (*green outlines*). Anti-MHC antibody-associated fluorescence is measured in the ring area. *Brown circles* show cells excluded because the secondary antibody fluorescence is below the cutoff. Further analyses (nuclear size and caspase 3 intensity) are made in MHC-positive cells (PDF 123 kb)
