# Supplementary figures and images for: Development of High Content Imaging Methods for Cell Death Detection in Human Pluripotent Stem Cell-Derived Cardiomyocytes
Source: J Cardiovasc Transl Res. 2012 Aug 16;5(5):593–604. doi: 10.1007/s12265-012-9396-1 (PMC3447146; doi:10.1007/s12265-012-9396-1)

Supplementary Figure 1.

**A**

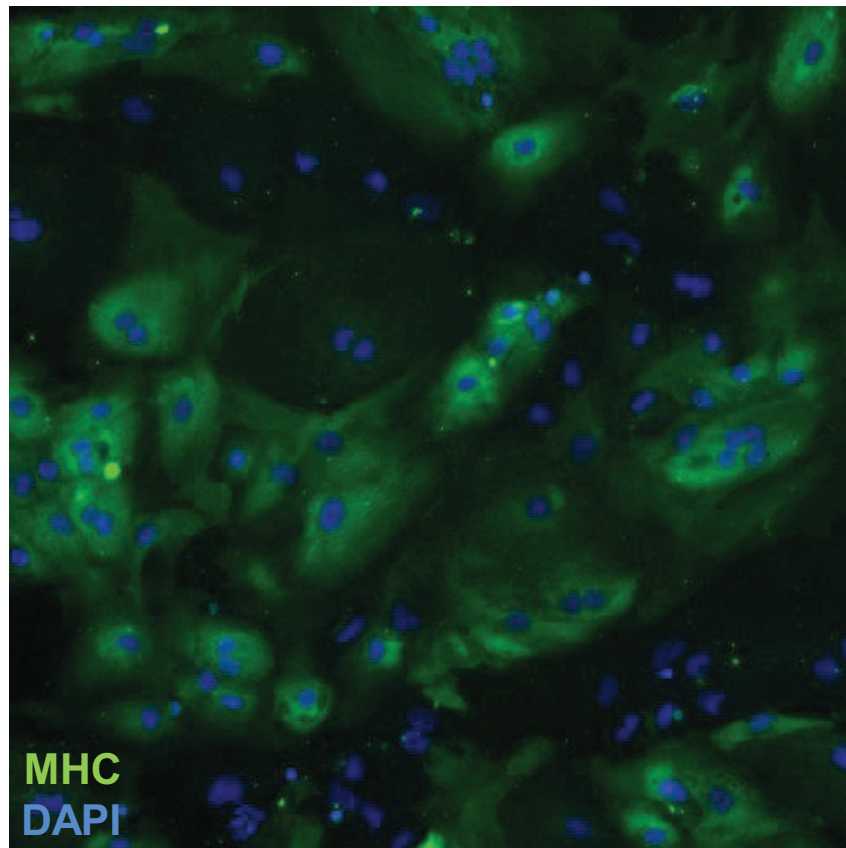

**B**

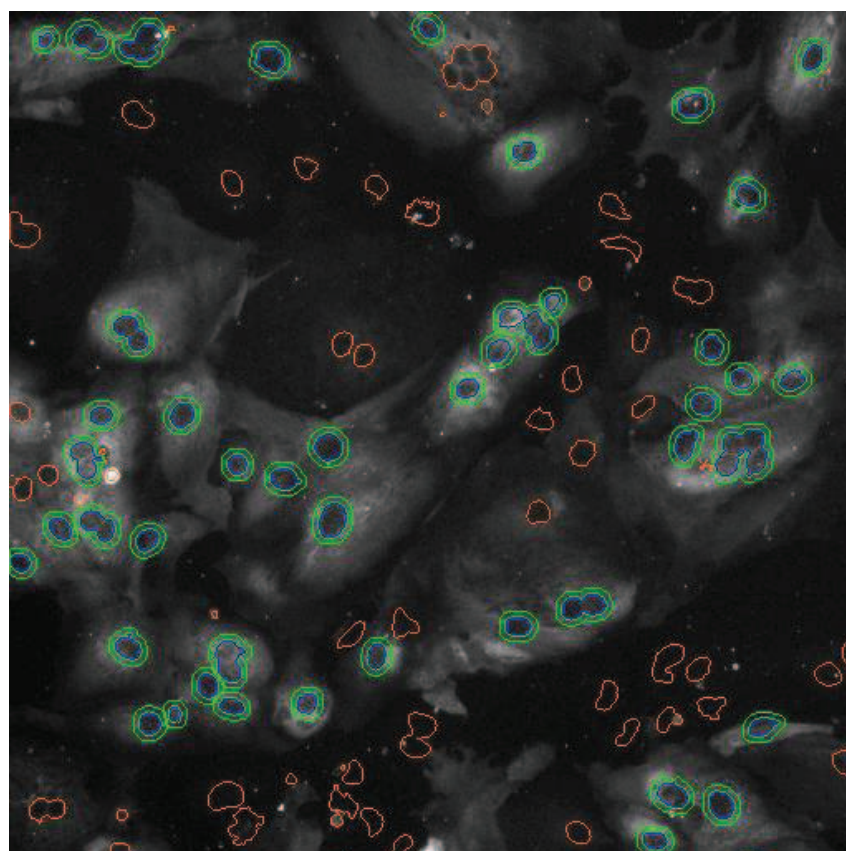

Supplement: Supplementary file 1 — Cellomics algorithm for the detection of MHC-positive hESC-CM. A DAPI (blue) identifies cell nuclei and MHC (green) defines cardiomyocytes in differentiated hESC cultures. B The bioassay algorithm detects each nucleus (blue inner outline for accepted nucleus) and thus delineates an inner region corresponding to the nucleus and an outer region called ring that applies from the peri-nuclear area (green outlines). Anti-MHC antibody-associated fluorescence is measured in the ring area. Brown circles show cells excluded because the secondary antibody fluorescence is below the cutoff. Further analyses (nuclear size and caspase 3 intensity) are made in MHC-positive cells (PDF 123 kb) [file 12265_2012_9396_MOESM1_ESM.pdf]
